# Influence of mobilization and weight bearing on in-hospital outcome in geriatric patients with hip fractures

**DOI:** 10.1051/sicotj/2019005

**Published:** 2019-02-28

**Authors:** Manuel Baer, Valentin Neuhaus, Hans Christoph Pape, Bernhard Ciritsis

**Affiliations:** Division of Trauma Surgery, Department of Surgery, University Hospital Zurich, University of Zurich Zurich Switzerland

**Keywords:** Hip fracture, Mobilization, Weight bearing, Complications, Mortality, In-hospital outcome

## Abstract

*Introduction*: Early recovery of mobilization after a fracture of the hip is associated with improved long-term ability to walk, lower complication rates, and mortality. In this context, early mobilization and full weight bearing are favorable. The aim of this study was (1) to analyze the influence of time between operation and first mobilization on in-hospital outcome and (2) the influence of early mobilization, full weight bearing, and ASA on pain, mobility of the hip, and ability to walk during the in-hospital phase of recovery.

*Methods*: This is a retrospective in-hospital study of 219 patients aged 70 years or older who were treated with surgery after a hip fracture. Data were collected by a review of medical records. The outcomes were mortality, complications, length of stay, and the Merle d’Aubigné score which evaluates pain, mobility of the hip, and ability to walk. Factors were sought in bivariate and multivariate analyses.

*Results*: A shorter time between operation and first mobilization was significantly associated with lower in-hospital mortality and complications. Early mobilization (within 24 h after the operation) and full weight bearing had no influence on pain, mobility of the hip, and ability to walk as well as length of stay in our cohort. Fracture type and treatment influenced mobility of the hip, while age as well as physical health status affected the ability to walk.

*Discussion*: Patients with femoral neck fractures, respectively after total hip arthroplasty, had less pain and showed better mobility of the hip and ability to walk during hospitalization than patients with trochanteric fractures; these results were irrespective of early vs. late mobilization and full vs. partial weight bearing. Foremost, a shorter time between operation and first mobilization is associated with lower complication and mortality rates.

## Introduction

The increase in life expectancy in the past few decades has led to a substantial increase in fragility fractures. Especially, hip fractures are common and serious injuries of elderly people that lead to loss of mobility and independency and result in significant socioeconomic consequences [1,2]. Elderly patients with hip fractures often present with comorbidities and frequently suffer complications during their hospital stay [3]. In addition, patients become further debilitated by pain, loss of mobility, and quality of life [1]. Although the main goals of the treatment of hip fractures are early mobilization and return to previous social activities, many patients never recover to their pre-fracture functional level [4,5].

Early recovery of mobilization has not only a significant impact on short-term results, such as lower complication rates and shorter length of stay, but also results in better long-term outcomes such as higher autonomy and reduced mortality [6–11]. Kristensen et al. showed that after adjustment for important pre-surgery variables, the mobility status at discharge is a predictor for long-term mortality [12]. Multiple factors (such as age, gender, social status, pre-fracture ambulatory level, and comorbidities) have been detected to have an influence on the functional outcome; however they can rarely be modified [13–17]. At the same time, potentially modifiable factors (full weight bearing, early mobilization, pain management) seem less studied to the best of our knowledge.

A multidisciplinary postoperative approach and early ambulation have been identified to increase functional outcome at discharge and enhanced performance in activities of daily living after hip fracture [3,7,18–21]. In this context, the effect of postoperative weight bearing is still controversial although full weight bearing seems to be favored [22–25].

The purpose of this study was (1) to analyze the influence of time between operation and first mobilization on in-hospital mortality as well as complications and (2) the association between early and late mobilization, full or partial weight bearing as well as American Society of Anesthesiologist Physical Status classification [26] (ASA) on pain, mobility of the hip, and ability to walk while controlling for type of fracture and treatment in geriatric patients with hip fractures.

## Materials and methods

### Study subjects

This retrospective observational study was approved by the local ethics committee (KEK-ZH-Nr. 2011-0382), and written informed consent was obtained from all patients.

Patients meeting the following criteria were included: (a) age over 70 years; (b) diagnosis of a femoral neck or trochanteric fracture treated with intramedullary nail (Gamma 3 Nail-Stryker^®^, Stryker Corporation, Kalamazoo, MI), total or partial hip prosthesis at our institution; (c) complete Merle d’Aubigné score [27] at discharge; (d) reported/documented ASA score, postsurgical treatment with either partial or full weight bearing, and time to first mobilization. Exclusion criteria were: (a) pathological fractures; (b) polytraumatized patients; (c) patients already hospitalized in a different department in our hospital; (d) non-operative treatment trial; (e) periprosthetic fracture; and (f) subtrochanteric fractures. A total of 294 patients with a hip fracture from a level I trauma center between 2011 and 2017 were reviewed. The final study cohort consisted of 219 patients.

### Surgical procedures

Patients receiving an intramedullary gamma nail were placed in supine position on the fracture table and closed reduction of the fracture was obtained. Traction was applied to the fracture, keeping the leg straight and then rotated 10–15° internally to complete the reduction of the fracture. Standard skin incisions were made after identifying the greater trochanter. A reamer was used to open the medullary canal, the nail was inserted (180 mm length and 130° neck-shaft-angle), and the femoral head lag as well as femoral shaft locking screws were placed using the targeting device. After removing the targeting device, the final position of the implant was verified using the image intensifier.

Total and partial hip prostheses were done in supine position on an orthopedic table with a mobile leg positioner. Arthrotomy was done by a minimal anterolateral access following osteotomy of the femoral neck and extraction of the femoral head. Following standard surgical procedures in hip prosthesis, the acetabulum was exposed followed by reaming and placing of the acetabular implant in the correct angle under image intensifier. After positioning the femur in hyperextension, adduction, and external rotation, the medullary canal of the proximal femur was opened and the stem was placed in both total and partial hip replacements. Using the image intensifier, positioning was controlled intraoperative.

### Review of medical records

An orthopedic medical doctor reviewed the medical records, anesthesiological and surgical reports, and reports from physiotherapists as well as nurses. The following parameters were collected: (a) gender; (b) age; (c) diagnosis; (d) type of treatment; (e) Merle d’Aubigné score with its three modalities; (f) ASA score; (g) full or partial weight bearing; (h) time interval between surgery and first mobilization; (i) complications; (j) mortality and (k) length of stay.

### Outcome parameters

For the first research question, the outcomes were (a) in-hospital mortality, (b) length of stay, and (c) occurrence of the following complications during hospitalization: deep vein thrombosis, pulmonary embolism, urinary tract infection, decubitus, gastroduodenal ulcers, pseudomembranous colitis, urosepsis, electrolyte dysregulation requiring treatment, delirium, renal insufficiency, pneumonia including aspiration pneumonia, heart and respiratory insufficiency, myocardial infarction, ileus, wound infection, transient ischemic attack or stroke, and death.

For the second research question (only patients discharged alive), the outcome was assessed at the end of hospitalization with the Merle d’Aubigné score which evaluated pain, mobility of the hip, and ability to walk on a scale of 0–6 for each item, where 0 indicated the worst and 6 the best outcome of the patient. The total minimum score was 0 and the maximum was 18 [27].

#### Pain

The seven subgroups were the following: 0: Pain is intense and permanent; 1: Pain is severe, even at night; 2: Pain is severe when walking and prevents activity; 3: Pain is tolerable with limited activity; 4: Mild pain when walking and the pain disappears with rest; 5: Pain is mild and inconsistent, normal activity is possible; 6: No pain.

#### Mobility of the hip

The seven subgroups were the following: 0: Ankylosis with bad position of the hip; 1: No movement possible; 2: Flexion <40°; 3: Flexion 40–60°; 4: Flexion 60–80°, patient can reach his foot; 5: Flexion 80–90° and abduction 15–30°; 6: Flexion >90° and abduction >30°.

#### Ability to walk

The seven subgroups were the following: 0: The patient is not able to walk; 1: The patient is bedridden or uses canes or crutches and personal help to go to the bathroom; 2: The patient can walk only with crutches or walking frame; 3: The patient can walk with canes; 3: The patient can walk with one cane for less than an hour, without a can only with much difficulties; 4: The patient can walk for a long period (>1 h) with a cane, short time without cane but with a limp; 5: The patient can walk without a walking aid but with a slight limp; 6: Normal walking abilities.

### Independent variables and confounders

Independent variables were age, gender, fracture type as well as fracture treatment. Confounders were the ASA score, full or partial weight bearing, and time interval between surgery and first mobilization.

#### ASA

An anesthesiologist preoperatively classified the patients undergoing surgery by the American Society of Anesthesiologist Physical Status classification (ASA) and documented it in the anesthesiologic report. The physical status were: Class I: a normally healthy patient; Class II: a patient with mild systemic disease; Class III: a patient with severe systemic disease that is not incapacitating; Class IV: a patient with an incapacitating systemic disease that is a constant threat to life; Class V: a moribund patient who is not expected to survive for 24 h with or without operation. No patient was classified ASA V. For statistical analysis, the patients were dichotomized into ASA I/II and ASA III/IV.

#### Weight bearing

The postoperative level of weight bearing was determined by the surgeon. Because not all patients were able to follow postsurgical weight bearing according to the surgeons’ prescription, there were discrepancies between surgical reports and reports from physiotherapists. The actual weight bearing performed by the patient was used as the postoperative level of weight bearing. Patients were mobilized either with full or partial weight bearing.

#### Mobilization

For the first research question, patients were classified into three categories by the time between the operation and first mobilization: (a) within 24 h, (b) between 24 and 48 h, (c) after 48 h.

For the second research question, early mobilization was defined as first mobilization of the patient within 24 h after surgery and late mobilization after 24 h.

### Statistical analysis

Standard descriptive statistical analyses were performed using SPSS software package (SPSS version 23, International Business Machines Corp., Armonk, NY). Continuous data were presented as mean values with standard deviation (SD), were examined for normal distribution by exact Kolmogorov–Smirnov test, and compared with either *t*-test or Mann–Whitney *U*-test. Differences in frequencies of categorical data were assessed by chi-squared test or Fisher’s exact test, depending on the number of expected cases per group. For the first research question, only variables, which were significant or nearly significant (*p* < 0.1) in bivariate analysis, were entered into a regression analysis with the main primary outcome complications. Due to a low number of deaths, no regression analysis was done for mortality. For the second research question, all independent and confounding variables were entered into a linear regression analysis with the main primary outcome parameters as well as the Merle d’Aubigné score, and length of stay.

A *p*-value of 0.05 was chosen as the significance cut-off level.

## Results

### Mortality

The overall in-hospital mortality rate was 7.3% (Table 1). In bivariate analysis, ASA as well as time between operation and first mobilization were associated with mortality (*p* = 0.001 and *p* < 0.001).

**Table 1:** Overview.

	Total		Mortality		Complications		Length of stay
	(*n* = 219)		(*n* = 16, 7.3%)		(*n* = 87, 39.7%)		(9.6 (±5.3))
	*n*	%	*n*	%	*n*	%	Days (±*SD*)
Gender							
Male	70	32	8	11.4	35	50	9.2 (±4.9)
Female	149	68	8	5.4	52	34.9	10.7 (±6.0)
Fracture type							
Trochanteric	157	72	11	7	63	40.1	9.3 (±5.3)
Femoral neck	62	28	5	8.1	24	38.7	10.7 (±5.1)
Fracture treatment							
Intramedullary nail	158	72	11	7	63	39.9	9.2 (±5.3)
Total hip replacement	35	16	2	5.7	13	37.1	10.8 (±5.2)
Partial hip replacement	26	11	3	11.5	11	42.3	10.6 (±5.1)
Mobilization							
Early	132	60	3	2.4	42	33.6	9.5 (±5.4)
Late	87	40	13	13.8	45	47.9	10 (±5.2)
Time to first mobilization							
<24 h	124	56	3	2.4	42	33.9	9.5 (±5.4)
24–48 h	77	35	6	7.8	33	42.9	10.5 (±5.3)
>48 h	18	8	7	38.9	12	66.7	7.5 (±3.8)
Weight bearing							
Full	153	70	8	5.2	58	37.9	9.4 (±5.3)
Partial	66	30	8	12.1	29	43.9	10.3 (±5.2)
ASA							
I/II	83	38	0	0	19	22.9	9.7 (±4.9)
III/IV	136	62	16	11.8	68	50	9.7 (±5.6)

ASA, American Society of Anesthesiologist Physical Status classification; *n*, numbers.

### Complications

The overall complications rate, including death, was 39.7%. Gender, ASA, and time between operation and first mobilization were associated with in-hospital complications (*p* = 0.033, *p* < 0.001, and *p* = 0.023). In multivariate analysis, a higher ASA score and longer time between operation and first immobilization (less than 24 h vs. longer than 48 h) were significant predictors for a higher complication rate (Table 2).

**Table 2:** Predictors for complications.

	Sig.	Exp (*B*)	95% CI	
			Lower	Upper
Gender (male)	0.128	1.599	0.874	2.929
ASA III/IV vs. I/II	0.001	2.946	1.568	5.534
Time to first mobilization				
<24 h vs >48 h	0.031	3.290	1.119	9.679
24–48 h vs >48 h	0.471	1.252	0.680	2.306

Dependent variable: Complications.

Predictors: (Constant), Gender, ASA, Time to first mobilization.

### Pain

The overall pain was 4.0 ± 0.9 (Table 3). Pain was significantly higher (lower score) in trochanteric fracture patients than in patients with femoral neck fractures (*p* < 0.001). The level of pain in patients treated with intramedullary nail was significantly higher compared to patients with total and partial hip replacements (*p* < 0.001 and *p* = 0.004). No predictors for pain were found in multivariate analysis (Table 4).

**Table 3:** Overview.

	Total	Pain	Mobility	Ability to walk	Merle d’Aubigné	Length of stay
	*n* = 203	4.0 (±0.9)	4.4 (±0.8)	1.5 (±0.9)	10.0 (±1.9)	9.8 (±5.1)
Age (y)	83 (7.1)	–	–	–	–	–
Gender						
Male	62 (30%)	4.1 (±1.0)	4.4 (±0.9)	1.6 (±0.9)	10.1 (±1.9)	9.2 (±4.7)
Female	141 (70%)	4.1 (±1.1)	4.4 (±0.8)	1.6 (±1.0)	10.1 (±2.0)	11.3 (±6.1)
Fracture type						
Trochanteric	146 (72%)	3.9 (±0.9)	4.3 (±0.8)	1.5 (±0.9)	9.7 (±1.7)	9.4 (±5.2)
Femoral neck	57 (28%)	4.6 (±1.1)	4.6 (±0.8)	1.8 (±1.0)	11.1 (±2.0)	11.0 (±5.1)
Fracture treatment						
Intramedullary nail	147 (72%)	3.9 (±0.9)	4.3 (±0.8)	1.5 (±0.9)	9.7 (±1.7)	9.4 (±5.2)
Total hip replacement	33 (16%)	4.7 (±1.1)	4.8 (±0.8)	2.2 (±0.9)	11.7 (±2.1)	11. 0 (±5.1)
Partial hip replacement	23 (11%)	4.6 (±1.2)	4.3 (±0.9)	1.4 (±0.9)	10.3 (±1.6)	11.3 (±5.2)
Mobilization						
Early	122 (60%)	4.1 (±0.9)	4.4 (±0.8)	1.5 (±0.9)	10.1 (±1.8)	9.4 (±5.3)
Late	81 (40%)	4.1 (±1.1)	4.4 (±0.9)	1.6 (±1.0)	10.1 (±2.1)	10.5 (±5.0)
Weight bearing						
Full	145 (71%)	4.1 (±0.9)	4.4 (±0.9)	1.6 (±0.9)	10.1 (±1.9)	9.5 (±5.2)
Partial	58 (29%)	4.1 (±1.1)	4.4 (±0.7)	1.6 (±0.9)	10.0 (±2.0)	10.8 (±5.2)
ASA						
I/II	83 (41%)	4.2 (±1.0)	4.4 (±0.8)	1.8 (±1.0)	10.4 (±2.0)	9.7 (±4.9)
III/IV	120 (59%)	4.0 (±1.0)	4.4 (±0.9)	1.4 (±0.9)	9.9 (±1.8)	10.0 (±5.4)

ASA, American Society of Anesthesiologist Physical Status classification; *n*, numbers.

**Table 4:** Predictors for pain.

	Unstandardized coefficients	Standardized coefficients	*t*	Sig.	95% CI	
	*B*	Beta			Lower	Upper
Age	0.002	0.012	0.167	0.867	−0.018	0.022
Gender	−0.017	−0.008	−0.113	0.910	−0.314	0.280
Fracture type	0.542	0.246	1.395	0.165	−0.224	1.309
Fracture treatment	0.139	0.095	0.543	0.588	−0.366	0.643
ASA	−0.154	−0.077	−1.067	0.287	−0.439	0.131
Mobilization	0.109	0.054	0.771	0.441	−0.170	0.387
Weight bearing	−0.076	−0.035	−0.499	0.619	−0.379	0.226

Dependent variable: Pain (0–6).

Predictors: (Constant), Weight bearing, Gender, Fracture type, ASA Low/High, Mobilization, Age, Fracture treatment.

### Mobility of the hip

The overall mobility was 4.4 ± 0.8. Mobility at the end of hospitalization was significantly better in femoral neck fracture patients than in patients with trochanteric fractures (*p* = 0.008). Patients treated with a total hip replacement were significantly better mobilized than patients after intramedullary nailing or partial hip replacement (*p* < 0.001 and *p* = 0.008). In multivariate analysis, fracture type and fracture treatment were significant: femoral neck as well as total hip replacement were associated with higher mobility of the hip (Table 5).

**Table 5:** Predictors for mobility.

	Unstandardized coefficients	Standardized coefficients	*t*	Sig.	95% CI	
	*B*	Beta			Lower	Upper
Age	−0.001	−0.005	−0.066	0.948	−0.018	0.017
Gender	0.036	0.020	0.275	0.783	−0.223	0.295
Fracture type	1.061	0.568	3.128	0.002	0.392	1.729
Fracture treatment	−0.535	−0.434	−2.399	0.018	−0.975	−0.095
ASA	0.051	0.030	0.401	0.689	−0.198	0.299
Mobilization	0.077	0.045	0.628	0.531	−0.166	0.320
Weight bearing	0.043	0.023	0.322	0.748	−0.221	0.307

Dependent variable: Mobility (0–6).

Predictors: (Constant), Weight bearing, Gender, Fracture type, ASA Low/High, Mobilization, Age, Fracture treatment.

### Ability to walk

The overall ability to walk was 1.5 ± 0.9. At discharge, femoral neck fracture patients and patients classified with ASA I/II had a significantly higher score for ability to walk (*p* = 0.016 and *p* = 0.011). Patients treated with total hip replacement had a better ability to walk than patients with an intramedullary nail or partial hip replacement (*p* < 0.001 and *p* = 0.002). In multivariate analysis, younger age as well as lower ASA score were found as predictors for ability to walk (Table 6).

**Table 6:** Predictors for ability to walk.

	Unstandardized coefficients	Standardized coefficients	*t*	Sig.	95% CI	
	*B*	Beta			Lower	Upper
Age	−0.033	−0.255	−3.606	<0.001	−0.051	−0.015
Gender	−0.058	−0.029	−0.423	0.673	−0.329	0.213
Fracture type	0.690	0.337	1.944	0.053	−0.010	1.389
Fracture treatment	−0.314	−0.232	−1.346	0.180	−0.775	0.146
ASA	−0.289	−0.155	−2.191	0.030	−0.549	−0.029
Mobilization	−0.068	−0.036	−0.528	0.598	−0.322	0.186
Weight bearing	−0.135	−0.066	−0.964	0.336	−0.411	0.141

Dependent variable: Ability to walk (0–6).

Predictors: (Constant), Weight bearing, Gender, Fracture type, ASA Low/High, Mobilization, Age, Fracture treatment.

### Merle d’Aubigné

The overall Merle d’Aubigné score at discharge was 10 ± 1.9. In bivariate analysis, patients with a) femoral neck fracture or b) with a total hip replacement were associated with a higher (better) Merle d’Aubigné score (*p* < 0.001 and *p* = 0.013). In multivariate analysis, femoral neck fracture was a significant predictor for a higher Merle d’Aubigné score (Table 7).

**Table 7:** Predictors for Merle d’Aubigné score.

	Unstandardized coefficients	Standardized coefficients	*t*	Sig.	95% CI	
	*B*	Beta			Lower	Upper
Age	−0.032	−0.119	−1.690	0.093	−0.070	0.005
Gender	−0.039	−0.009	−0.137	0.891	−0.597	0.520
Fracture type	2.293	0.541	3.133	0.002	0.849	3.736
Fracture treatment	−0.711	−0.254	−1.476	0.142	−1.660	0.239
ASA	−0.393	−0.101	−1.443	0.151	−0.929	0.144
Mobilization	0.118	0.030	0.444	0.657	−0.406	0.642
Weight bearing	−0.168	−0.040	−0.583	0.561	−0.737	0.401

Dependent variable: Merle d’Aubigné (0–18).

Predictors: (Constant), Weight bearing, Gender, Fracture type, ASA Low/High, Mobilization, Age, Fracture treatment.

### Length of stay

The overall length of stay was 9.8 ± 5.1 days. Female patients, femoral neck fractures, partial weight bearing, and treatment with total or partial hip replacements were significantly associated with a longer in-hospital stay (*p* = 0.015, *p* = 0.021, *p* = 0.027, *p* = 0.038). In multivariate analysis, gender was the only significant predictor for length of stay (Table 8).

**Table 8:** Predictors for length of stay (in days).

	Unstandardized coefficients	Standardized coefficients	*t*	Sig.	95% CI	
	*B*	Beta			Lower	Upper
Age	−0.026	−0.035	−0.472	0.637	−0.133	0.081
Gender	1.945	0.173	2.413	0.017	0.355	3.534
Fracture type	0.540	0.047	0.259	0.796	−3.567	4.647
Fracture treatment	0.510	0.067	0.373	0.710	−2.192	3.213
ASA	−0.111	−0.011	−0.143	0.886	−1.639	1.417
Mobilization	−0.720	−0.068	−0.951	0.343	−2.212	0.772
Weight bearing	1.087	0.095	1.324	0.187	−0.532	2.707

Dependent Variable: Lengh of stay (in days).

Predictors: (Constant), Weight bearing, Gender, Fracture type, ASA Low/High, Mobilization, Age, Fracture treatment.

## Discussion

Early mobilization and full weight bearing in geriatric patients with hip fractures are usually associated with a faster and uneventful recovery. The aim of this study was (1) to analyze the influence of time between operation and first mobilization on in-hospital mortality as well as complications and (2) the influence of early mobilization, full weight bearing, and healthy physical status on in-hospital pain, mobility of the hip, and ability to walk in geriatric patients with hip fractures. First, a shorter time between operation and first mobilization was significantly associated with lower in-hospital mortality and complications. Second, early mobilization and full weight bearing had no influence on pain, mobility of the hip, and ability to walk as well as length of stay in our cohort. Fracture type and treatment mainly influenced mobility of the hip, and age as well as physical health status impacted the ability to walk.

There are several limitations of the study. First, it is a retrospective, observational study; second, scores were obtained by a review of medical records and reports; third, only in-hospital follow-up was analyzed; and last, the limited number of patients.

### Mortality

The in-hospital mortality rate in our study is 7.3%. This is consistent with international studies which showed in-hospital mortality rates between 3 and 10% [13,16,28–31]. In addition, ASA score has been previously found to have an influence on mortality after a fracture of the hip [14,23,32]. In our cohort, a short time to first mobilization is associated with a lower mortality rate. This is consistent with Siu et al.; they showed that prolonged immobility was associated with higher mortality rates at six months [33]. Still, the association of early mobilization and lower mortality rate seems not to be very surprising, since early ambulation has been shown to have less complication and, therefore, in connection lower mortality. In our cohort, there is a threshold of 24 h between operation and first mobilization.

### Complications

We found less complication in patients mobilized in the first 24 h after operation than in patients mobilized later than 24 or 48 h. This is comparable with previous studies [6–9]. At the same time, lower ASA and female gender were associated with lower complication rates. This has been shown in many different studies, which analyzed preoperative patient characteristics with in-hospital or long-term outcome [16,34]. The question remains the causality.

### Pain

The level of pain was higher in trochanteric fractures and patients treated with an intramedullary nail. This is consistent with international studies [18,35,36]. In contrast to these studies, we found no negative influence of pain on the ability of early mobilization [18,35,36]. This may be due to our orthogeriatric concept of intense physiotherapy and pain management. At least, early mobilization and full-weight bearing did not increase the level of pain.

We found no negative predictors for pain; however we only analyzed several factors. Besides fracture type and fracture treatment, a short time to fracture treatment, the mental health status or depression have been identified as a predictor for pain in different studies [1,7]. The focus in treating hip fracture patients must be an adequate pain management, especially in patients with trochanteric fractures, and a short time to fracture treatment.

### Mobility of the hip

Ninety percent of our patients reached a mobility score of four or higher at the end of the hospital stay. In other words, only 10% were not able to reach their feet. Fracture type and treatment were predictors for hip mobility, but early mobilization and full weight bearing had no influence on hip mobility.

Different assessment tools have been used to evaluate the functional outcome after surgery of the hip. In this study we used the Merle d’Aubigné score, which contains the mobility of the hip score. Since nearly all patients, independent of our confounders, had the same mobility of the hip score, this mobility of the hip score seems inadequate to be used for this purpose. The cumulative ambulation score (CAS) seems to be more useful in the clinical context. It evaluates specific motion sequences like getting in and out of bed or sit to stand from a chair over the first three postoperative days.

### Ability to walk

In this study group, only 3% of the patients were able to walk without crutches for a short distance. Forty one percent of the patients were able to walk with crutches. This seems to be low compared with other studies reporting 53–80% of patients reaching pre-injury functional level [28,30,31]. Early mobilization and full weight bearing did not have any effect on the mobility of the hip and ability to walk until discharge. Nevertheless, since it is common in Switzerland for elderly patients with hip fracture to enter a rehabilitation facility or nursing home before reaching their preoperative state, the ability to walk should be compared after rehabilitation.

Full weight bearing seems to be favored and associated with a better functional outcome [22,24]. In addition, orthogeriatric patients might have problems to follow a partial weight bearing protocol depending on their overall health status. In this context, a higher ASA-Score and a higher age were the only predictors for a lower ability to walk in our study population. This is consistent with other studies [13,14,23,32].

## Conclusions

This study emphasizes the importance of early mobilization after a surgical hip procedure to reduce complications and death. It showed also reproducible risk factors for a negative in-hospital outcome, such as higher age and higher ASA score. Early mobilization and weight bearing did yet not have any short-term effect on pain, mobility of the hip, and ability to walk in our cohort. Fracture type and fracture treatment mainly influenced the in-hospital functional outcome.
